# Supplementary material for: Causal Relationship between Aging and Anorexia Nervosa: A White-Matter-Microstructure-Mediated Mendelian Randomization Analysis
Source: Biomedicines. 2024 Aug 16;12(8):1874. doi: 10.3390/biomedicines12081874 (PMC11351342; doi:10.3390/biomedicines12081874)
Supplement: Supplementary file 1 [file biomedicines-12-01874-s001.zip › Supplementary Figure/Figure S8/Leaveoneout plot of MR analysis for TL to ubm-b-1734.pdf]

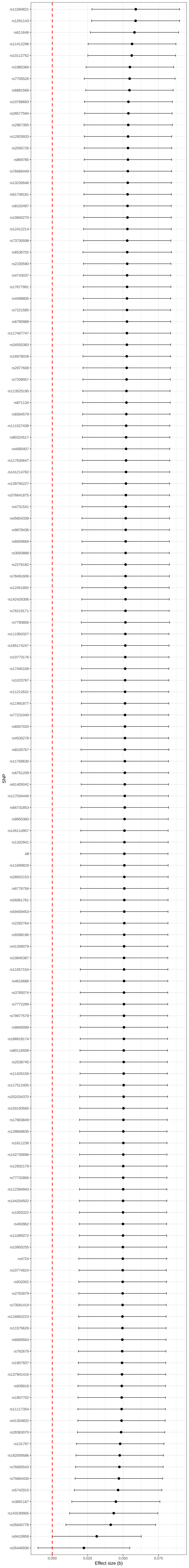

rs11584821
rs1291143
rs611646
rs11412296
rs10112752
rs1985369
rs7705526
rs6881568
rs10768683
rs28577594
rs2967355
rs12925933
rs2056726
rs869785
rs76666449
rs13230646
rs61748181
rs8102497
rs10840270
rs12412214
rs73730598
rs6536702
rs2230590
rs4743037
rs17677991
rs4498805
rs7221585
rs6790988
rs117407747
rs34550383
rs16978028
rs2977608
rs7209057
rs113525195
rs871134
rs6584579
rs111527438
rs80324517
rs4695407
rs117630647
rs141214782
rs139795227
rs376641875
rs4731541
rs45604339
rs9878436
rs6659669
rs3093888
rs2276182
rs78491606
rs12451892
rs142426306
rs76219171
rs7790856
rs111950327
rs185174247
rs10773176
rs17445108
rs1023767
rs11212631
rs11991877
rs77231040
rs6007020
rs4530278
rs8105767
rs11769630
rs6751209
rs61405042
rs117034449
rs66731853
rs9955360
rs145114957
rs1332941
All
rs11699829
rs28502153
rs6776756
rs56061761
rs59409453
rs2282764
rs9398196
rs41269079
rs10845387
rs11557154
rs4616688
rs3785074
rs7772289
rs79977570
rs9940099
rs188918174
rs80116508
rs2538745
rs11426156
rs117512405
rs202034370
rs150150565
rs17803849
rs139669835
rs1611236
rs142730698
rs12932179
rs77732866
rs112394943
rs144204502
rs1003322
rs450962
rs11085072
rs10905255
rs4724
rs10774624
rs932002
rs2763979
rs73581419
rs116863223
rs11579626
rs6669563
rs762679
rs1957937
rs137901416
rs939916
rs1907702
rs11117354
rs41304832
rs28363070
rs131797
rs182059586
rs76065543
rs75664430
rs5742915
rs3891167
rs143190905
rs35640778
rs9419958
rs35446936
SNP
0.000
0.025
0.050
0.075
Effect size (b)
